# Interaction of Nanodiamonds with Water: Impact of Surface Chemistry on Hydrophilicity, Aggregation and Electrical Properties

**DOI:** 10.3390/nano11102740

**Published:** 2021-10-16

**Authors:** Pietro Aprà, Lorenzo Mino, Alfio Battiato, Paolo Olivero, Sofia Sturari, Maria Carmen Valsania, Veronica Varzi, Federico Picollo

**Affiliations:** 1 Physics Department, University of Torino, Via Pietro Giuria 1, 10125 Torino, Italy; pietro.apra@unito.it (P.A.); paolo.olivero@unito.it (P.O.); sofia.sturari@edu.unito.it (S.S.); veronica.varzi@unito.it (V.V.); federico.picollo@unito.it (F.P.); 2 “Nanostructured Interfaces and Surfaces” (NIS) Inter-Departmental Centre, University of Torino, Via Quarello 15/a, 10135 Torino, Italy; mariacarmen.valsania@unito.it; 3 National Institute of Nuclear Physics, Section of Torino, Via Pietro Giuria 1, 10125 Torino, Italy; battiato@to.infn.it; 4 Department of Chemistry, University of Torino, Via Pietro Giuria 7, 10125 Torino, Italy

**Keywords:** nanodiamonds, surface chemistry, hydrophilicity, IR spectroscopy, Raman spectroscopy, nanoparticle aggregation

## Abstract

In recent decades, nanodiamonds (NDs) have earned increasing interest in a wide variety of research fields, thanks to their excellent mechanical, chemical, and optical properties, together with the possibility of easily tuning their surface chemistry for the desired purpose. According to the application context, it is essential to acquire an extensive understanding of their interaction with water in terms of hydrophilicity, environmental adsorption, stability in solution, and impact on electrical properties. In this paper, we report on a systematic study of the effects of reducing and oxidizing thermal processes on ND surface water adsorption. Both detonation and milled NDs were analyzed by combining different techniques. Temperature-dependent infrared spectroscopy was employed to study ND surface chemistry and water adsorption, while dynamic light scattering allowed the evaluation of their behavior in solution. The influence of water adsorption on their electrical properties was also investigated and correlated with structural and optical information obtained via Raman/photoluminescence spectroscopy. In general, higher oxygen-containing surfaces exhibited higher hydrophilicity, better stability in solution, and higher electrical conduction, although for the latter the surface graphitic contribution was also crucial. Our results provide in-depth information on the hydrophilicity of NDs in relation to their surface chemical and physical properties, by also evaluating the impacts on their aggregation and electrical conductance.

## 1. Introduction

Nanodiamonds (NDs) are attracting ever-increasing interest for their appealing physical, chemical, and optical properties for a broad range of technological applications. The most widely employed techniques for their synthesis are represented by the detonation of explosive carbon-based compounds (e.g., trinitrotoluene and hexogen) [1], laser ablation [2], milling of high-pressure high-temperature (HPHT) diamond microcrystals [3], and chemical vapor deposition (CVD) [4]. Initially, due to the extreme hardness and the low friction coefficient of diamond, NDs were mainly employed in tribology [5]. Over time, they turned out to be interesting for a much wider variety of applications, particularly in the context of nanocomposites, sensing, quantum emitters, and energy storage and conversion [6]. Moreover, their low toxicity, biocompatibility, and the possibility of tailoring their surface chemistry make NDs good candidates for biomedical applications—such as drug delivery [7], tissue engineering [8], and bioimaging [9]—thanks to their fluorescence, characterized by high photostability and resistance to bleaching and quenching phenomena. The latter property of diamond is ascribable to the presence of a large variety of lattice defects that can act as “color centers”. One of the most documented and natively present defects of the synthesis processes is the nitrogen-vacancy (NV) defect [10], consisting of a substitutional nitrogen atom near to a vacancy defect. This system possesses two optically active states—namely, the negatively charged NV^−^ center, with a zero-phonon line (ZPL) spectrally located at 638 nm, and the neutral-charge-state NV^0^ center, with a ZPL emission spectrally located at 575 nm. The wide phonon band provides a stable emission at 600–800 nm, with an excitation window at 500–600 nm. Moreover, the electronic structure of the NV^−^ center shows peculiar spin-dependent radiative transitions that can be exploited as a high-sensitivity magnetometer and thermometer, by means of optically detected magnetic resonance (ODMR), with appealing prospects in high-sensitivity and high-resolution sensing applications [11,12,13,14,15].

Depending on the synthesis method and on the subsequent treatments, NDs display different surface functional groups (–OH, –COOH, –C=O, etc.), as well as outer shells constituted by disordered sp^2^ and sp^3^ carbon phases of different thickness, ranging from 0.1 to 1 nm [16,17,18], which also affect their optical properties. An important issue in applications such as electrochemical reactions, catalysis [19,20] and biological/biomolecular interfacing [21] is the control of the interface between the surface and water or aqueous solutions. Thus, a deep understanding of the surface chemistry is essential to shed light on this interaction, in terms of both adsorption of environmental moisture and behavior in solution. When adsorbed from the environment, water molecule interaction occurs on the so-called “primary adsorption centers”, such as oxygen-containing functional groups, which allow the formation of hydrogen bonds [22]. Subsequently, adsorbed water molecules act as secondary adsorption centers, leading to the formation of a continuous water film that fills the micropores between the nanoparticles.

Several previous works were already focused on the study of this phenomenon. Fourier-transform infrared (FTIR) spectroscopy was extensively employed to analyze the surface chemistry of NDs [23], also following diverse treatments such as air [18] or ozone purification [24], hydrogenation [25], hydroxylation [26], and functionalization processes with specific functional groups, biomolecules, and drugs [27,28,29,30]. By means of FTIR measurements, Ji et al. provided solid information concerning the effects of water adsorption on detonation NDs modified under different conditions, evaluating both the level of water adsorbed from the environment and the speed of the adsorption process itself [31]. In the same work, temperature-programmed desorption (TPD) was also carried out, evidencing a clear water-release peak around 100–120 °C. Stehlik et al. [32] employed thermogravimetric analysis (TGA) to evaluate the weight loss due to desorbed water under heating, while Petit et al. [33] studied the water hydrogen bond network in aqueous ND dispersions by using infrared, Raman, and X-ray absorption spectroscopies.

The effect of surface chemistry on the interaction with adsorbed water also has a substantial influence on the electrical properties of NDs, whose control is relevant in the context of ND-based capacitors [34] and humidity sensors [35,36]. Indeed, adsorbed water molecules allow conduction to take place via the Grotthuss mechanism. This phenomenon occurs with the hopping of a proton from a hydroxonium ion to an adjacent water molecule, involving breaking and reforming of the covalent and hydrogen bonds. This implies the formation of a new H_3_O^+^ ion which, in turn, transfers the proton to another water molecule, according to the chain reaction: H_2_O + H_3_O^+^ → H_3_O^+^+ H_2_O. This mechanism results in a high electrical conductivity which, as seen by Denisov et al. [22], rises further when increasing the amount of adsorbed water. It has also been shown by Piña-Salazar et al. that the conductivity substantially drops upon heating NDs in vacuum, since the treatment causes the removal of adsorbed water [37].

Surface termination control is even more essential in the context of the water interaction of NDs in solutions and aqueous environments. Dynamic light scattering (DLS) and Z-potential analysis are generally employed to assess dispersibility and stability in solution. Indeed, low hydrophilicity is correlated with high aggregation levels, and since proper dispersibility is of paramount importance in the development of nanomedical and biosensing devices, over time, many modifications and functionalization protocols have been developed to deal with it [38,39,40,41].

Nevertheless, a better understanding of the interaction of NDs with water, as well as systematic studies including milled NDs and differently sized nanocrystals, are still necessary. In this work, three batches of NDs with different dimensions (nominal average sizes varying from 5 nm to 500 nm) were investigated following their modification via thermal annealing and oxidation processes. We compared the results of a variety of characterization techniques, with a particular focus on variable-temperature diffuse-reflectance infrared Fourier-transform (DRIFT) spectroscopy, DLS, and electrical conductivity analysis, providing helpful information on the influence of surface chemistry on hydrophilicity, hydration, dispersibility, and electrical conductance. Scanning electron microscopy (SEM) and Raman/photoluminescence (PL) spectroscopy were also performed to combine the above-described outcomes with structural and optical information, respectively.

## 2. Materials and Methods

### 2.1. Nanodiamonds Samples and Thermal Treatments

Three ND batches were employed in this study, namely:

- Detonation nanodiamonds (DNDs) from Adamas (Research Triangle Park, NC, USA), with 5 nm nominal primary particle size;
- Nanodiamonds produced by Element Six™ (Harwell Oxford, UK) from the milling of HPHT type-Ib single crystals (milled nanodiamonds (MNDs)):
  ○ Micron+ with particle size varying in the 0–250 nm range (small milled nanodiamonds (s-MNDs));
  ○ Micron+ with particle size varying in the 0.5–1 μm range (large milled nanodiamonds (l-MNDs)).

NDs were investigated both as-received and after their processing with thermal annealing in nitrogen flow and a subsequent air oxidation treatment. A tubular furnace was employed to this effect, in order to guarantee a proper gas flow and an efficient removal of released species during the process [42,43]. The annealing process was performed with the purpose of reorganizing the disordered sp^2^/sp^3^ phases [44] and graphitizing the amorphous external layers, while fully preserving the diamond phase. Moreover, the oxygen-free environment causes a chemical reduction of the ND surface. To avoid unwanted structural and chemical degradation of the samples, the definition of an optimal processing temperature is essential. On the one hand, the nitrogen environment prevents the chemical degradation of diamond that would occur at temperatures above 500 °C in the presence of oxygen [45]; on the other hand, despite the inert atmosphere, it is necessary to maintain the temperature below 900 °C to avoid the graphitization [46] and conversion of the NDs to carbon onions [47]. Thus, the annealing treatment was carried out at 800 °C for 2 h. Afterwards, in order to selectively remove the superficial graphitic layers and to decorate the ND surface with carbonyl and carboxyl groups, the air oxidation process was performed at 450 °C for 8 h, thus keeping below the diamond air degradation temperature.

### 2.2. Diffuse-Reflectance Infrared Fourier-Transform (DRIFT) Spectroscopy

DRIFT spectra were collected in diffuse reflectance mode using a Bruker Equinox 55 spectrometer (Bruker Optik, Ettlingen, Germany) equipped with a Spectra Tech DRIFT accessory (model 0030-011) and a mercury cadmium telluride detector. This setup allows the acquisition of spectra in a controlled atmosphere or under vacuum (i.e., residual pressure < 10^−3^ mbar), at a variable temperature from room temperature up to 400 °C. The samples were characterized by averaging 64 interferograms at 2 cm^−1^ spectral resolution. The reflectance data were successively converted in pseudo-absorbance: A = −log R, where R is the measured reflectance.

### 2.3. Electrical Measurements

Current–voltage (I–V) characteristics were acquired to assess the effects of thermal treatments on NDs in terms of their electrical conduction properties. NDs were heated on a thermal plate at 120 °C for 15 min to remove the previously adsorbed water, thus “resetting” their initial condition. Subsequently, the samples were exposed to a controlled environment with temperature (21 ± 1) °C and humidity h_1_ = (32 ± 2)% or h_2_ = (61 ± 2)%. After 15 h, NDs were loaded into a polyamide cylindrical measurement chamber (depth t = (2.85 ± 0.05) mm and diameter d = (7.00 ± 0.05) mm) and closed with two aluminum plates working both as closing clamps and as electrodes. Two springs ensured the sealing of the cell and the contact of the electrodes with the powder, applying constant and uniform pressure. A Keithley 6487 electrometer (Tektronix, Beaverton, OR, USA), featuring an output impedance < 100 Ω and optimized for resistances ranging from 50 Ω to 10^12^ Ω, was employed to record I–V curves in the range −20 V/+20 V, with steps of 1 V, in a two-electrode configuration. The resistance (R) values were calculated for each cell to compare the electrical conduction properties of the different specimens.

### 2.4. SEM/TEM Microscopy

An Inspect F™ scanning electron microscope (SEM) with a field emission gun was adopted in secondary electron (SE) detection mode to investigate the morphological features and the size distributions of the s-MND and l-MND samples. The system operated at 5 kV acceleration voltage in high-vacuum conditions (i.e., ~10^−6^ mbar pressure), allowing for ~10 nm spatial resolution. The imaged samples were prepared by dispersing the NDs in isopropyl alcohol, with a concentration of ~0.1 mg mL^−1^. The solutions were then processed with an Elmasonic S15H (Singen, Germany) sonicator (35 W ultrasonic power) for 15 min, and droplets of the suspensions were deposited on Si substrates. The NDs under analysis were not metal-coated; although leading to possible charging effects, this choice was made in order to avoid any modification of the NDs morphology. The distribution of the dimensions was assessed using Fiji software—an ImageJ-based open-source image processing package—as suggested in a previous work [48]. After enhancing their contrast, the micrographs were converted into binary, and the “watershed” feature from the BioVoxxel Toolbox was applied to separate contiguous particles. The application of the automatic particle analysis provided data on the projected areas of the nanocrystals, which were reconverted to diameters by assuming a spherical shape of the nanoparticles. Finally, by merging the data from 2–3 micrographs for each sample, the particle size histograms were built by applying a suitable binning.

Due to their small nominal size, DND analysis was conducted with a JEOL 3010 UHR TEM microscope (JEOL Ltd., Tokyo, Japan), operating at an acceleration voltage of 300 kV. Samples were prepared by depositing the same solutions employed for the SEM specimen preparation over a lacey-carbon copper grid. Because of the grainy images of the primary particles, their sizes were estimated by the user in a non-automated procedure.

### 2.5. DLS Analysis

To study the aggregation level and stability of NDs in a liquid environment, the samples were investigated via DLS measurements. A Malvern Instruments Zetasizer Nano ZS (Malvern, UK) was employed to this effect, operating with a 4 mW He−Ne 633 nm laser (173° scattering angle), and by setting the refractive index of diamond (2.4). ND size analysis was conducted on 1 mg mL^−1^ suspensions in distilled water, while analysis of both size [49] and Z-potential [50] was performed in a 0.5 mM NaCl water solution with a 50 μg mL^−1^ concentration of NDs. The suspensions were prepared from as-received, annealed, and annealed + oxidized DND, s-MND, and l-MND samples. The use of a minimal saline concentration was necessary in order to guarantee a proper electric field propagation along with the measurement cell during Z-potential analysis [51]. Before every acquisition, the solutions were processed for 15 min with the above-mentioned sonicator.

### 2.6. Raman and Photoluminescence Spectroscopies

Raman and PL spectroscopies were employed to obtain information about the structural and optical properties of NDs, by identifying the presence of diamond, graphitic, and amorphous carbon phases, and the fluorescence arising from NV centers. The samples were prepared by dispersing the NDs powder in isopropyl alcohol and depositing a uniform ~0.2 mm thick layer on a silicon substrate. Raman and PL spectra of all of the samples were acquired using a Horiba Jobin Yvon HR800 Raman microspectrometer (Horiba Italia S.r.l., Torino, Italy) equipped with a CCD detection system with a Peltier cooling system (−70 °C) and a continuous Nd-YAG 532 nm excitation laser. The effective power density on the sample position was 0.01 mW µm^−2^, which allowed the quick collection of spectra while avoiding the self-heating effects that typically affect nanoparticle analysis [27]. In the measurements, the integration time was set to 20 s, and 3 repeated acquisitions were averaged to improve the signal statistics. The employed 20× objective allowed the detection of an averaged signal coming from a relatively wide sample area (10 × 10 µm^2^) and ~3 μm in confocal depth, while the 600 lines mm^−1^ diffraction grating provided a spectral resolution of ~3 cm^−1^.

## 3. Results and Discussion

**Figure 1:**
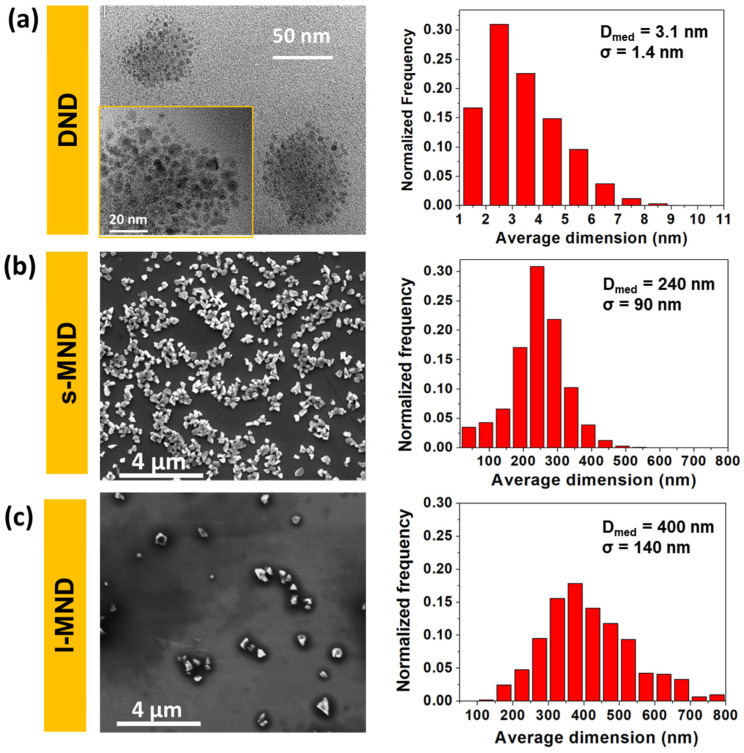
TEM (DNDs (**a**)) and SEM (s-MNDs (**b**) and l-MNDs (**c**)) images and derived size distributions.

Figure 1 shows the TEM (DNDs) and SEM (s-MNDs and l-MNDs) micrographs of the pristine samples and their derived size distributions. The nanocrystals are characterized by sharp and irregular shapes. While MNDs are well dispersed, DND primary particles are observable mainly in aggregates of 20–100 nm in diameter. Median size values are 3.1 nm for DNDs, 240 nm for s-MNDs, and 400 nm for l-MNDs. Standard deviations of the distributions are particularly large (σ _DND_: ~1.4 nm, σ _s-MND_: ~90 nm and σ _l-MND_: ~140 nm), suggesting the use of fractionation processes when, according to the application context, a narrower dispersion is needed [52,53].

The selected particles allow the investigation and comparison of chemical and physical properties over a broad range of size distributions, providing a comprehensive overview of the influence of the NDs dimensions on the interaction with water.

**Figure 2:**
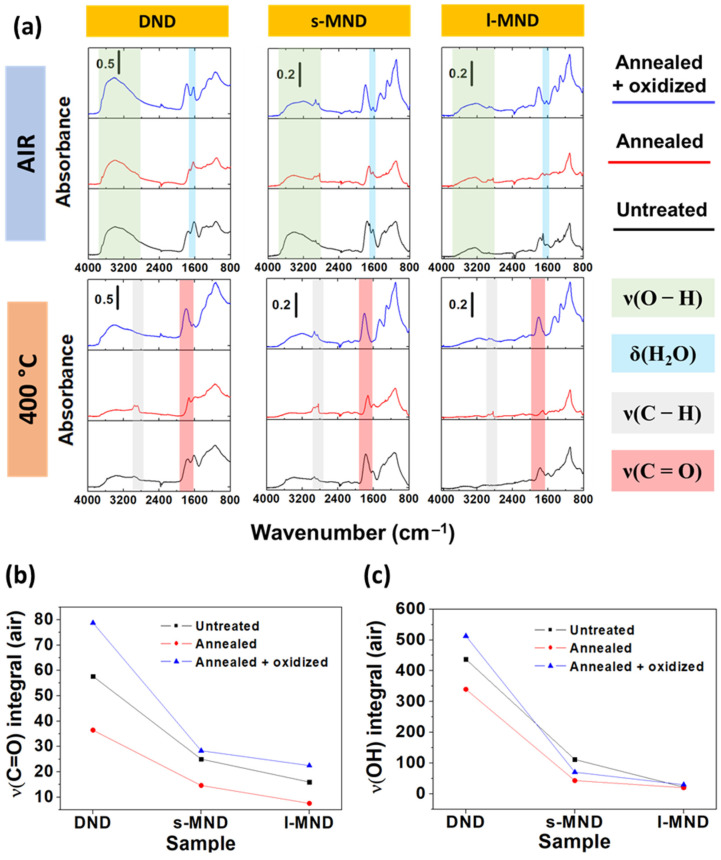
(**a**) DRIFT spectra of DNDs, s-MNDs, and l-MNDs at different processing steps, in room conditions and outgassed at 400 °C; (**b**) ν(C=O) band integral of the samples at the different processing steps, in room conditions; (**c**) ν(O–H) band integral of the samples at the different processing steps, in room conditions.

DRIFT analysis was performed to provide a detailed understanding of the surface chemistry of NDs at the different processing steps, by suitably identifying the functional groups. Figure 2a reports the spectra collected in an air environment and after outgassing at 400 °C of DND, s-MND, and l-MND samples. For each sample type, the measurements were carried out at the different processing steps—namely, untreated, annealed and annealed + oxidized. The spectra of the untreated NDs are characterized by the presence of a broad absorption in the 3650–3000 cm^−1^ range, ascribed to the ν(O–H) stretching modes of hydrogen-bonded water molecules, which also give rise to the band of the δ(H_2_O) bending mode at 1630 cm^−1^ [31,54]. The very weak signals in the 3000–2800 cm^−1^ spectral region, overlapped with the previously described broad OH band, are associated with ν(C–H) stretching. The strong components in the 1800–1720 cm^−1^ range are due to the vibrations of C=O groups, which can be assigned to different surface moieties, including carboxylic acids, esters, lactones, acid anhydrides, etc. [23]. In the low-wavenumber region, in which the signals are more overlapped, the presence of C–O vibrational modes between 1200 and 1000 cm^−1^ is visible [26]. Although the different NDs particle sizes and aggregations can complicate the quantitative analysis of diffuse-reflectance IR spectra, the absolute intensity of the IR signals for the different materials is clearly related to the particle dimensions (i.e.,: to the specific surface area) and, as expected, follows the order DNDs > s-MNDs > l-MNDs.

After the annealing process in reducing conditions, a decrease in the C=O groups and a general shift of their signals toward lower wavenumbers are observed, suggesting the disappearance of a relevant fraction of the oxygenated compounds. The subsequent surface rearrangement leads to a parallel increase in the number of C–H species. This is highlighted by the rise of two peaks at 2950 cm^−1^ and 2886 cm^−1^, which can be assigned to the asymmetric and symmetric stretching modes of CH_3_ groups, respectively [55]. These signals are particularly evident in the spectra collected from the samples upon outgassing at 400 °C (Figure 2a). This can be explained on the basis of the fact that, upon the above-mentioned treatment, the NDs surface is cleared of adsorbed molecules and, therefore, the interference of molecular water is minimized [56,57]. It is worth noting that, after the annealing process, in the MND samples, a sharp component grows at 2837 cm^−1^, which can be associated with CH_2_ groups.

The following oxidation step decreases again the presence of C–H species, consistently with what was observed in previous studies [31], while increasing the concentration of C=O groups. The results of the integration (after proper baseline subtraction) of the corresponding IR band reported in Figure 2b highlight how the amount of oxygenated terminations is increased with respect to the untreated samples.

The above-described surface modifications also have a strong impact on the affinity of the surface towards the water, as indicated by the evident variation in the intensity of the δ(H_2_O) and ν(O–H) signals (see Figure 2a,c). In particular, the hydrophilicity follows the order annealed + oxidized > untreated > annealed, in agreement with the easier formation of hydrogen bonds on surfaces richer of oxygen-containing groups.

**Figure 3:**
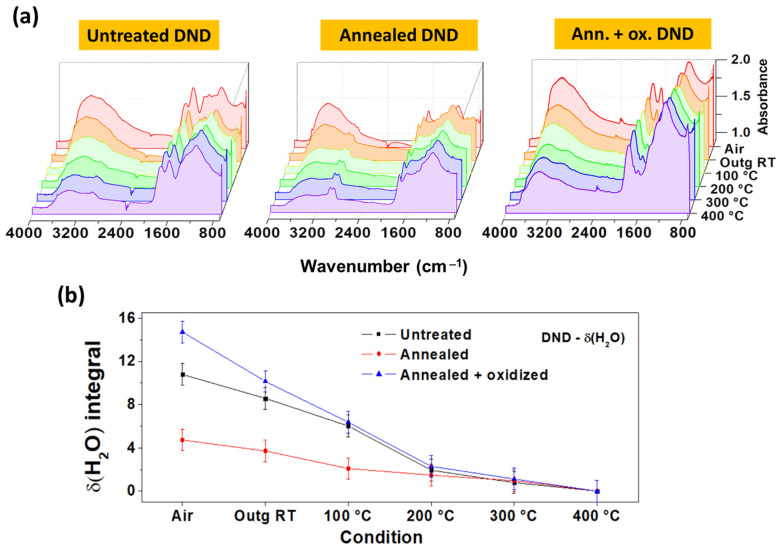
(**a**) DRIFT spectra of untreated, annealed, and annealed + oxidized DNDs as a function of the applied temperature; (**b**) δ(H_2_O) band integral as a function of the acquisition condition for untreated, annealed, and annealed + oxidized DNDs.

Finally, the progressive surface dehydration under vacuum at increasing temperatures was monitored by performing in situ DRIFT measurements, which allowed us to distinguish between hydroxyls and molecular water contributions, owing to their different temperature stability [58]. A representative set of the acquired spectra is reported in Figure 3a, and the results of the integration of the signal across the δ(H_2_O) band are shown in Figure 3b. From these results, it is possible to conclude that H_2_O molecules, which initially form a surface multilayer in wet air, are almost completely desorbed at 200 °C. Conversely, OH groups are still partially bonded to the surface up to 400 °C, as confirmed by the presence of the ν(O–H) band even at the highest treatment temperature—particularly visible in the annealed + oxidized DND samples.

**Figure 4:**
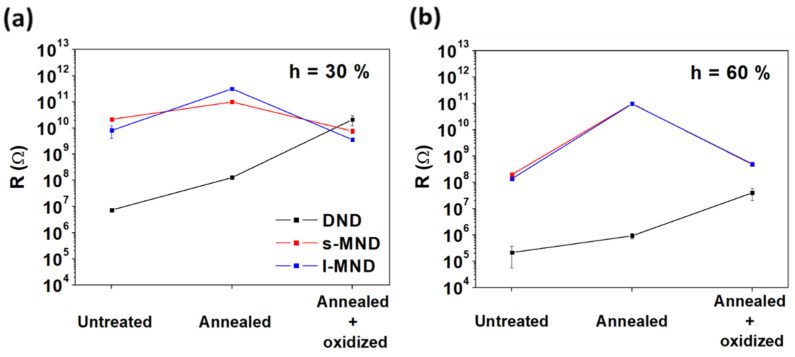
Electrical resistance of NDs at the different processing steps after 15 h exposure to 30% (**a**) and 60% (**b**) humidity.

As discussed in the introduction, water adsorption can influence the surface conductivity of NDs. Electrical measurements were performed both immediately after heating and 15 h after the exposure to the controlled environment. In the first case, all samples exhibited no measurable conductance, with currents smaller than 1 pA at 20 V, thus being below the range of sensitivity of the measuring setup and suggesting the complete desorption of water. After exposure to 30% relative humidity conditions for 15 h, both MND samples presented a similar resistance, ranging between 10^10^ and 10^11^ Ω, while DNDs were more conductive (Figure 4a). In general, consistently with their higher water adsorption capacity observed in DRIFT analysis, oxidized and untreated NDs presented a higher conductance with respect to the annealed samples, with the peculiar exception of oxidized DNDs, whose resistance was settled above 10^10^ Ω. Upon increasing the relative humidity to 60% (Figure 4b), untreated and annealed DND samples showed a two-orders-of-magnitude reduction in resistance. Similar variations were observed for untreated and oxidized MNDs, while the annealed MNDs did not show any increase in Grotthuss conduction, remaining stuck at resistance values of 10^11^ Ω. Although showing an unexpectedly high resistance in comparison with the other processing steps, oxidized DNDs exhibited the most substantial decrease (~3 orders of magnitude) in resistance when passing from the lower to the higher hydration conditions (i.e., from 30% to 60% relative humidity). This evidence implies a considerably higher water adsorption upon increasing relative humidity. Together with the intense δ(H_2_O) absorption shown by DRIFT spectra (Figure 3b), this result suggests that the reason behind this apparent inconsistency may be found in the structural properties of the different NDs types.

**Figure 5:**
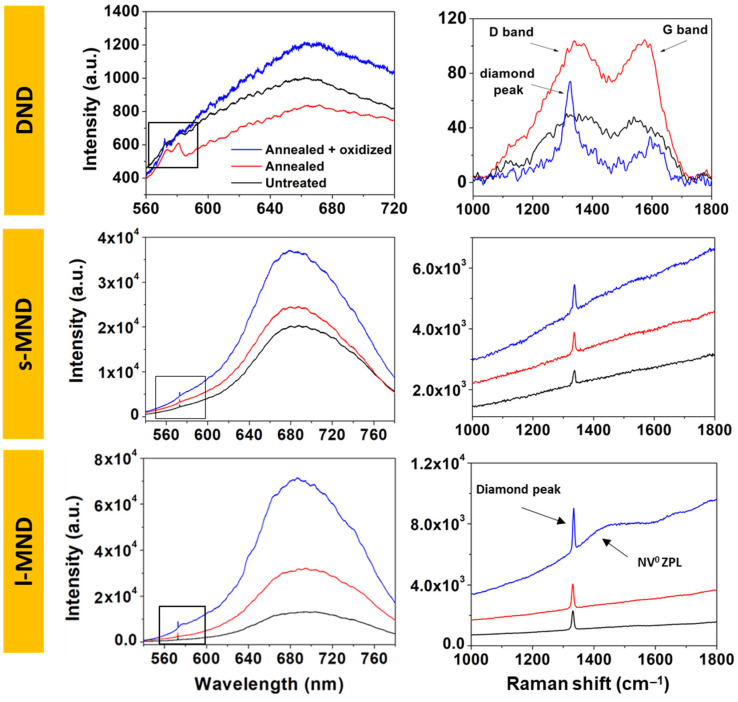
Photoluminescence spectra (**left**) and zoom of the main Raman features (**right**) of untreated, annealed, and annealed + oxidized DNDs, s-MNDs, and l-MNDs. A baseline was subtracted from the Raman spectra of DNDs to allow for better identification of D- and G- band signals.

To evaluate this effect, Raman and photoluminescence spectroscopies were employed to characterize the samples. Figure 5 shows the resulting spectra from DNDs, s-MNDs, and l-MNDs at each processing step. Untreated DND spectra are characterized by two main Raman features—namely, the D-band at 1360 cm^−1^, indicative of disordered and amorphous carbon phases, and the G-band at 1580 cm^−1^, which is typical of defective graphite-like structures. No first-order Raman diamond peak was evident for the untreated and annealed DNDs, while a noisy signal was visible in the annealed + oxidized samples. These observations are consistent with the very small dimensions of the primary DND particles. Indeed, the relevant amount of sp^2^-defective shells and their higher Raman scattering cross-section (*r*) with respect to that of diamond phases (*r*_sp3_ = 9 × 10^−7^ cm^−1^ sr^−1^; *r*_sp2_ = 5 × 10^−5^ cm^−1^ sr^−1^ [59]) should result in a negligible Raman signal from the latter phase. Nevertheless, to further ensure the presence of a core diamond phase, Raman analysis was performed on annealed + oxidized DNDs using 442 nm laser excitation, since the shorter excitation wavelength enables an easier detection of the sp^3^-bonded phases of the Raman signals [60]. Indeed, the resulting spectrum shows the characteristic diamond peak (see Figure S1 in the Supplementary Materials).

For all the 532 nm excitation Raman spectra of the MND samples, the D- and G-bands are no longer observable, while the first-order diamond Raman peak appears around 1332 cm^−1^. The latter results more intense for l-MNDs compared to s-MNDs, due to the higher diamond bulk contribution and the lower surface graphitic contaminations, in accord with the minor stresses generated in the milder grinding synthesis procedures of larger-sized nanocrystals. The thermal annealing process increases the G-band intensity in DND samples, consistently with the graphitization of the amorphous carbon components of the NDs’ outer layers. Conversely, the subsequent air oxidation results in the chemical etching of such sp^2^ phases and, thus, in a significant decrease in the intensity of their Raman features. The PL emission across the 580–780 nm range, which is mostly ascribable to the formation of NV centers, is very faint in DND samples, with no significant variations upon thermal processing. Again, this can be attributed to the very low sp^3^ diamond content potentially hosting the color centers. Conversely, s- and l-MNDs exhibit a strong increase in the fluorescence yield, particularly upon oxidation, with the appearance of two recognizable shoulders at ~575 nm and ~638 nm, corresponding to the zero-phonon lines (ZPLs) of the NV^0^ and NV^−^ color centers, respectively. This effect is attributable to the oxidation-induced removal of the PL-quenching contribution of the defective outer layers, as highlighted in a previous work [61]. On the basis of this evidence, we infer that, contrary to what was observed in MND samples, the rich graphitic content of DND samples plays a crucial role in their measured resistance values. In particular, while the high electrical conductance of sp^2^ components was significant in untreated and annealed DNDs, in oxidized samples this contribution disappeared.

**Figure 6:**
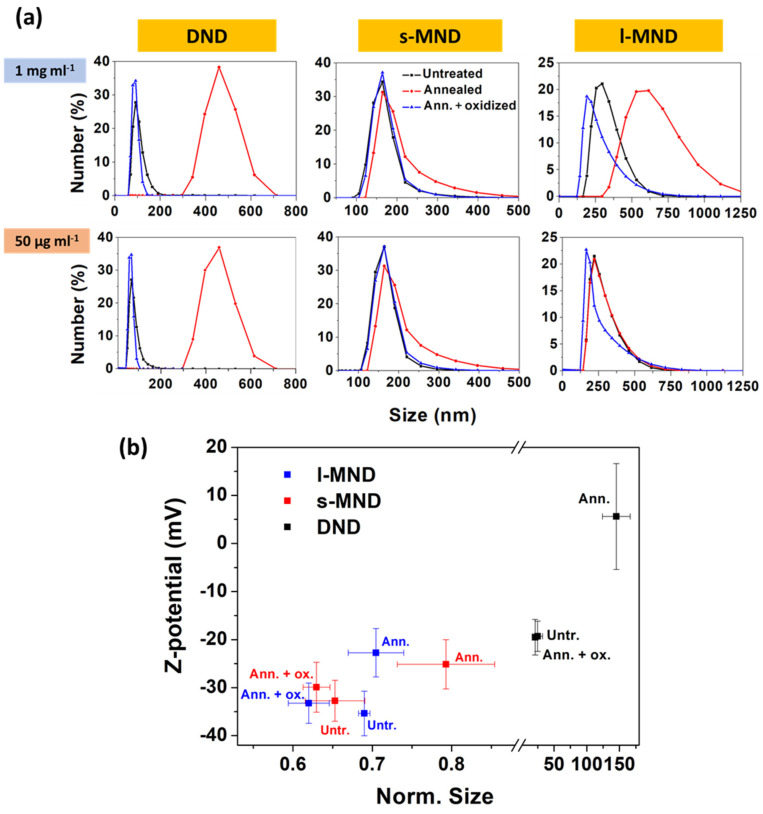
(**a**) DLS size distribution by number and (**b**) Z-potential as functions of the average size of the distributions by number, normalized by the median dimension of the primary nanoparticles.

The surface chemical characterization of NDs also has crucial importance for a careful assessment of their behavior in aqueous solution. The dispersion level and the stability of the ND suspension at 1 mg mL^−1^ in distilled water (i.e., a condition in which ND aggregation is more likely) and at 50 µg mL^−1^ in NaCl 0.5 mM water solution (i.e., to favor ND dispersion) were evaluated by means of DLS analysis. Figure 6a shows the size distributions of both suspensions, by number. In general, due to aggregation phenomena, annealed NDs present higher size values, suggesting poor dispersibility in water. This is interpreted on the basis of their higher hydrophobicity, as confirmed by DRIFT analysis. On the other hand, the higher water affinity of the oxygenated chemical species decorating the surface of the untreated and oxidized NDs facilitates their solubility and reduces their aggregation. Both s-MND and l-MND samples are characterized by size values compatible with the dimensions of the primary particles, while all DND samples presented a severe aggregation level that was particularly pronounced upon thermal annealing. This is not unexpected, because of the wider interaction surface of DND samples. Moreover, it has been shown that, during detonation synthesis, the single NDs can interact to form strongly bound 20 nm aggregates [62], which can, in turn, form multiple clusters up to 1 micron in size that are difficult to segregate under hydrophobic surface conditions.

In the first approximation, we can consider ND nanoparticles forming spherical aggregates, and define an aggregation parameter by dividing the peak value of the size distributions by the median size of the NDs, as identified by the TEM/SEM micrographs for DNDs, s-MNDs, and l-MNDs (3.1, 240, and 400 nm, respectively). These results are presented along with Z-potential analysis in Figure 6b. Stronger surface potentials were clearly correlated with better dispersibility. Indeed, a higher surface charge should determine stronger repulsions between particles, thus avoiding aggregation and instability. The surface potential of untreated and oxidized samples was higher than that of annealed NDs, due to the presence of oxygen-containing functional groups, consistently with the results of DRIFT analysis [63]. DND samples—and particularly the annealed ones—exhibit a very low surface potential which, together with their higher exposed surface, explains their poor stability in solution. Moreover, it is worth noting that, although the DRIFT spectra of DND samples showed apparently richer amounts of oxygen-terminated groups with respect to MND samples, the related absorption bands should be interpreted by taking into account the markedly higher surface-to-volume ratio of DND samples. This consideration, combined with the differences in terms of surface Z-potential, may suggest a denser oxygen-terminated surface for MND samples compared to DND samples [64].

## 4. Conclusions

In the present work, nanodiamonds with different size distributions were investigated in terms of water interaction, both from environmental moisture and in solution. In particular, 5 nm detonation nanodiamonds (DNDs), along with 240 nm and 400 nm milled nanodiamonds (s-MNDs and l-MNDs, respectively) were analyzed both before and after annealing in a nitrogen environment and subsequent thermal oxidation in air. DRIFT analysis allowed the individuation of the main functional groups (mainly C=O, C–H, and O–H) and the evaluation of the water affinity of the nanoparticles, by varying the applied temperature up to 400 °C in situ. Because of the easier hydrogen bond formation, oxidized DNDs showed the stronger water interaction, followed by untreated and annealed samples. MNDs exhibited a similar behavior, but with an overall lower intensity of the IR signals, including that of water-related absorption features, due to their lower surface-to-volume ratio. Electrical measurements were conducted at 30% and 60% humidity conditions to evaluate the relevance of the Grotthuss mechanism occurring in the presence of adsorbed water. The results were consistent with the expectations provided by DRIFT spectra, excepted for DND samples, whose conductivity was markedly affected by the surface graphitic contribution. Indeed, as evidenced by Raman spectroscopy, surface oxidation was effective in removing these defective layers, causing higher electrical resistance and the disappearance of G-band Raman features in DNDs. On the other hand, the Raman spectra of MND samples did not show relevant graphite-related vibrations, although, following oxidation, a strong increase in the photoluminescence yield was observed, probably due to the removal of residual PL-quenching phases from the surface. Untreated DNDs are thus preferable when a higher electrical conductance is required, taking advantage of both the surface graphitic content and the significant environmental water adsorption due to their good hydrophilicity, while annealed MNDs are recommended when needing to avoid both these effects and to guarantee an insulating interface.

Finally, DLS and Z-potential analysis highlighted mutually consistent hydrophilicity profiles in water solution. In general, because of their richer content of oxygen-containing groups, oxidized and untreated NDs were better dispersible and more stable compared to annealed NDs. MNDs could easily be dispersed in aqueous solution, while DNDs showed a higher aggregation level and weaker surface charge, thus being less appealing for drug delivery and biomedical applications, where deagglomeration and good stability in solution are essential. Although the large surface-to-volume ratio of DND primary nanoparticles allowed for the overall higher water adsorption reflected in the DRIFT spectra, the surface potential analysis suggested a denser oxygenated surface for MNDs compared to DNDs.

These results provide useful insight into the connection between NDs surface chemistry and their hydration efficiency, with the systematic characterization of differently sized and surface-modified NDs samples. Moreover, these experimental results may be helpful for future NDs applications, especially in those contexts where their water adsorption properties, their stability in solution, or their electrical conductivity represent key issues.

## Data Availability

The data presented in this study are available on request from the corresponding author.

## Supplementary Materials

The following are available online at https://www.mdpi.com/article/10.3390/nano11102740/s1, Figure S1: Raman spectrum of annealed + oxidized DND under 422 nm laser excitation.
