# Supplementary material for: Interaction of Nanodiamonds with Water: Impact of Surface Chemistry on Hydrophilicity, Aggregation and Electrical Properties
Source: Nanomaterials (Basel). 2021 Oct 16;11(10):2740. doi: 10.3390/nano11102740 (PMC8538990; doi:10.3390/nano11102740)
Supplement: Supplementary file 1 [file nanomaterials-11-02740-s001.zip › nanomaterials-1416436-supplementary.pdf]

Supporting Information

Interaction of Nanodiamonds with Water: Impact of Surface Chemistry on Hydrophilicity, Aggregation and Electrical Properties

Pietro Aprà [1,2,3], Lorenzo Mino [2,4,*], Alfio Battiato [3], Paolo Olivero [1,2,3], Sofia Sturari [1], Maria Carmen Valsania [2], Veronica Varzi [1,2,3] and Federico Picollo [1,2,3]

1 Physics Department, University of Torino, via Pietro Giuria 1, 10125 Torino, Italy; pietro.apra@unito.it (P.A.); paolo.olivero@unito.it (P.O.); sofia.sturari@edu.unito.it (S.S.); veronica.varzi@unito.it (V.V.); federico.picollo@unito.it (F.P.)
2 "Nanostructured Interfaces and Surfaces" (NIS) Inter-Departmental Centre, University of Torino, via Quarello 15/a, 10135 Torino, Italy; mariacarmen.valsania@unito.it
3 National Institute of Nuclear Physics, Section of Torino, via Pietro Giuria 1, 10125 Torino, Italy; battiato@to.infn.it
4 Department of Chemistry, University of Torino, via Pietro Giuria 7, Torino, Italy
* Correspondence: lorenzo.mino@unito.it

Due to the low signal-to-noise ratio for the first-order diamond Raman peak in the spectra acquired on DND samples, we conducted a new measurement with a Renishaw inVia Raman Microscope under 422 nm He-Cd laser excitation, with 80× focusing objective. Spectral resolution was 1 cm^{-1} (grating 2600 lines mm). Figure. S1 shows the resulting Raman spectrum acquired for annealed + oxidized DND. Main observable features are the first-order diamond Raman peak at 1328 cm^{-}1 and the G-band around 1600 cm^{-1}. The wide signal surrounding the diamond peak is ascribable to D-band. The spectrum evidences the presence in the investigated DND samples of a diamond core phase, which was less evident in the spectra shown in the main article acquired under 532 nm laser excita tion. This is due to the higher Raman scattering cross-section of these phases with respect to diamond [1]. Conversely, this discrepancy is reduced as the excitation wavelength decreases, thus allowing the detection of the diamond Raman peak [2]

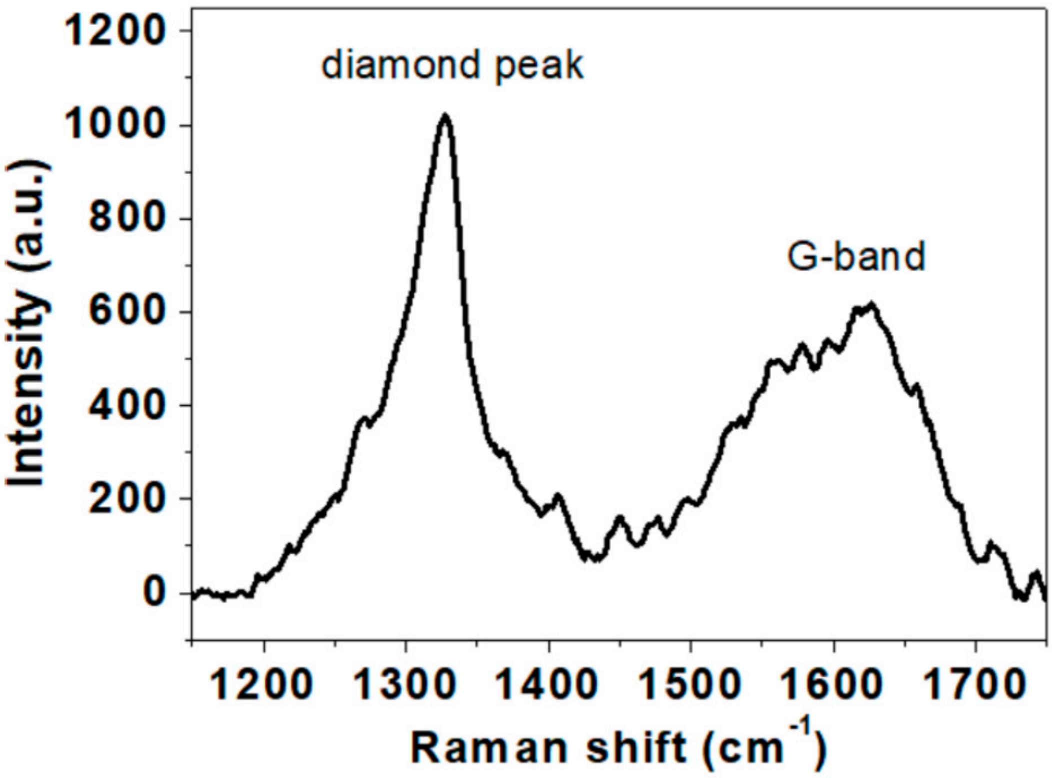

Figure S1: Raman spectrum of annealed + oxidized DND under 422 nm laser excitation

References

1. Zaitsev, A.M. Optical Properties of Diamond; Springer Berlin Heidelberg: Berlin, Heidelberg, 2001; ISBN 978-3-642-08585-7.

2. Birrell, J.; Gerbi, J.E.; Auciello, O.; Gibson, J.M.; Johnson, J.; Carlisle, J.A. Interpretation of the Raman spectra of ultrananocrystalline diamond. Diam. Relat. Mater. 2005, *14*, 86–92, doi:10.1016/j.diamond.2004.07.012.
